# Community-based maternal, newborn, and child health surveillance: perceptions and attitudes of local stakeholders towards using mobile phone by village health volunteers in the Kenge Health Zone, Democratic Republic of Congo

**DOI:** 10.1186/s12889-018-5186-2

**Published:** 2018-03-05

**Authors:** Mulamba Diese, Albert Kalonji, Bibiche Izale, Susie Villeneuve, Ngoma Miezi Kintaudi, Guy Clarysse, Ngashi Ngongo, Abel Mukengeshayi Ntambue

**Affiliations:** 1 Center for Applied Research and Development, Kinshasa, Democratic Republic of the Congo; 2 Santé Rurale (SANRU), Kinshasa, Democratic Republic of the Congo; 3 UNICEF Democratic Republic of Congo, Kinshasa, Democratic Republic of the Congo; 4 UNICEF New York, New York, USA; 5 Epidemiology and Maternal, Newborn and Child Unit, School of Public Health, University of Lubumbashi, Lubumbashi, Democratic Republic of Congo

**Keywords:** Maternal, newborn, and child health surveillance, Mobile technology, Community, perceptions, attitudes

## Abstract

**Background:**

In early 2016, we implemented a community-based maternal, newborn, and child health (MNCH) surveillance using mobile phones to collect, analyze, and use data by village health volunteers (VHV) in Kenge Health Zone (KHZ), in the Democratic Republic of Congo (DRC). The objective of this study was to determine the perceptions of households, attitudes of community health volunteers, and opinions of nurses in Health center and administrative authorities towards the use of mobile phones for MNCH surveillance in the rural KHZ in the DRC.

**Methods:**

We used mixed methods combining phenomenological and descriptive cross-sectional study. Between 3 and 24 March 2016, we collected the data through focus group discussions (FGD) with households, and structured interviews with VHV, local health and administrative authority, and nurses to explore the perceptions on MNCH surveillance using mobile phone. Data from the FGD and interviews  were analyzed using thematic analysis techniques and descriptive statistics respectively.

**Results:**

Health issues and services for under-five children were well known by community; however, beliefs and cultural norms contributed to the practices of seeking behavior for households. Mobile phones were perceived as devices that render quick services for people who needed help; and the community’s attitudes towards the mobile phone use for collection of data, analysis, and use activities were good. Although some of community members did not see a direct linkage between this surveillance approach and health benefits, majority believed that there would be better MNCH services with the use of mobile phone. In addition, VHV will benefit from free healthcare for households and some material benefits and training. The best time to undertake these activities were in the afternoon with mother of the child, being the best respondent at the household.

**Conclusion:**

Health issues and services for under-five children are well known and MNCH surveillance using mobile phone by VHV in which the mother can be involved as respondent is accepted.

**Electronic supplementary material:**

The online version of this article (10.1186/s12889-018-5186-2) contains supplementary material, which is available to authorized users.

## Background

The Democratic Republic of the Congo (DRC) has the fifth highest burden of under-five deaths globally, with 391,000 deaths in 2013 [1]. The most recent Demographic and Health Survey [2] reported an under-five mortality rate (U5MR) of 104 per 1000 live births. Most of these deaths are due to diseases of poverty and conditions that are both preventable and treatable, such as neonatal causes, malaria, diarrhea, pneumonia, and malnutrition [1]. The DRC is a very large country with an area of 2,345,409 km^2^ and an estimated population of 70 million. Most of the population lives in villages, where transportation and communication networks are undeveloped, and the socio-economic situation and state of health systems are weak [3].

The Ministry of Health has recently launched a national child survival acceleration plan 2013–2016 with integrated community case management (iCCM), as a major strategy for scaling up life-saving child survival interventions [4]. This plan promotes four main strategic shifts; one of which is community engagement in creating demand and utilizing child survival interventions through the reactivation of local village health committees (VHV) called, “*Cellules d’Animation Communautaire”*. These VHVs are volunteers who conduct head-counts and regularly update vital statistics; control usage of insecticide-impregnated bed nets and manage cases of fever (malaria) with ACTs; assess respiratory infections through respiratory rate timers; administer amoxicillin; distribute and replenish family kits of Oral rehydration serum, zinc, and sprinkles; and promote key family practices for children under 5 years of age. Currently, the VHVs haphazardly provide reports to the local health center (HC) nurses on the consumption of commodities and number of households visited [5]. These reports are either verbal or written on piece of paper.

DRC has recently introduced a District Health Information System 2 (DHIS2) to replace its old system that was outdated. The DHIS2 is as central reporting system that collects health statistics for the entire country. It is a web-based management information system across health zones (HZ) to collect, validate, analyze, and present aggregate statistical data in the health facilities at HZ level. The data reported by this system come from monthly paper-based reports from health facilities and do not provide community disaggregated nor real-time data. Furthermore, this system does not provide the real picture of health issues in the community because of limited access to healthcare for most of population. Even for those who access to it, a large amount of information about them is missing because of lack or weak record keeping practices at health facilities.

Another challenge of the health information system in DRC is the weak community-based surveillance system. This weak system is partly due to limited communication between health facilities and the population. Even where this system exists, data reported to HZ are not disaggregated per community to trigger local corrective actions. The only regular health reporting system that is working is a weekly epidemiological report for epidemic surveillance. This system is paper-based reports and the completeness and promptness of these reports by HZ is subject to transport challenges between health facilities and HZ management offices.

A real-time data report system can be achieved with the use of mobile technology at community level [6–8]. Despite the successful use of mobile phone for disease surveillance by community members [6–8], and as data management tools in many settings [9], its utilization in DRC is still very limited. In the context of epidemiological surveillance, its use remains limited to the sending of SMSs for weekly epidemiological records. There is however, no system for the centralization of these SMS within the health system and the coherence between the data transmitted by SMS and those that are captured at DHIS2 at HZ levels.

The objective of this study was to determine the perceptions of households, attitudes of community health volunteers, and opinions of nurses in HCs and administrative authorities towards the use of mobile phones for MNCH surveillance in the rural Kenge Health Zone (KHZ) in the DRC.

## Methods

### Setting

We conducted this study in the rural KHZ, located 300 km east of Kinshasa, in the Kwango province, DRC. It consists of 125 villages with a total population estimated at 248,659 [10]. This number of inhabitants varies from one village to another; it is 21 for small villages and 5000 for the largest. As per HZ office, the number of children under 5 years is estimated to 44,759. The KHZ is divided into 20 health areas [10].

### Study population, *Study and data collection*

The number of subjects included in the study depended on each methodological approach. We used the mixed methods combining phenomenological [11] and descriptive cross-sectional approaches. Phenomenological approach (qualitative) [12] focused on the following aspects: community-based under-five children health services; community practices regarding the seeking behavior for health services for under-five children; community’s attitude towards the use of the mobile phone; community’s participation in mobile phone data collection, analysis, and use activities and community’s expectations regarding data collection on MNCH at the household level. The cross-sectional approach (quantitative) aimed to determine the willingness, commitment, capacity and competencies, and motivation of VHV for the MNCH surveillance using mobile phone. We collected data between March 3rd and 24th 2016 using two techniques: focus group discussions and structured interviews using questionnaire.

### Qualitative approach

We conducted five focus group discussions (FGD) with 19 households’ heads in each, representing a health catchment area. The duration of FGD was determined by the saturation of the responses. This saturation was reached when two discussions were not providing additional information [13, 14]. The discussions took place in a hut assigned by the village chief in a selected village (Additional file 1 on list of selected villages for households FGD). The average duration of the discussions was 45 min, which were conducted in “Kiyaka,” the local dialect using the discussion guide topics (Table 1). Most FGD participants were young men and women. The women’s mean age was 30 years, and they had experience of childbirth and completed primary education. All of them were housewives involved in some income-generating activities, such as agriculture and selling small goods. Men comprised 15% of the FGD participants. They were involved in services, such as agriculture, hunting, fishing, and small-scale trading. Both men and women had lived in the current villages for some time ranging between 10 to 30 years.

**Table 1:** Discussion topics for the FGD with the households

Nbr	Discussion topics for FGD with households
1	General knowledge of the community-based under-five children health services
2	Community practices regarding the seeking behavior for health services for under-five children
3	Community’s attitude towards the use of mobile phone and collection of data
4	Community’s participation in mobile phone data analysis and use of corrective actions
5	Community’s expectations regarding data collection on MNCH at the household level

The FGDs were organized by gender to ensure the homogeneity and balance during the discussions [15]. Two weeks prior to the study, two people of native “Kiyaka” speakers were trained as FGD moderators to facilitate the proceedings. All moderators had never previously been involved in the activities of the child survival program.

Before starting the FGD, the moderators explained the discussion proceedings and read the study information from the consent form to the participants. Moreover, personal and professional demographic data of the participants were collected prior to the discussion. During the FGD, one moderator took notes, while the other guided the discussion using the discussion guide topics [16, 17]. These moderators switched roles after each discussion topic. Each FGD proceedings were supervised by a study team member. Discussions were fully recorded on a digital audio recorder. Each FGD was transcribed in Microsoft Word by two study assistants and then revised by the study investigators prior to analysis [13].

### Quantitative approach: Structured interview (using questionnaire)

Structured interviews were conducted with all village leaders and VHV; *n* = 190), one nurse, one administrative and health Local Authority from each of the five Health catchment areas were included in the study. The participant information sheet was read and explained to the participants.

The one-to-one interviews with village leaders and VHV members were held during the day at the village chief’s hut and lasted 20 min. Questions in the data collection tool (Table 2) were initially developed in French and then translated to Kiyaka.

**Table 2:** Data collection tool for the interview with the village leaders and volunteers

Nbr	Items
1	What are the health issues of under-five children in your village?
2	What are health services for under-five children in your village?
3	What is the current community-based surveillance for under-five children health services?
4	Do you know how to use mobile phone applications?
5	If you receive a mobile phone for MNCH surveillance-related services, what other needs will you use it for?
6	Would you want to participate in the MNCH surveillance using mobile phones?
7	Can you use a mobile phone to collect data at households? Yes/No
8	Can you analyze MNCH data collected with a mobile phone application? Yes/No
9	What type of incentives can you receive to undertake MNCH surveillance?
10	How many households can you visit per day?
11	Who is the appropriate respondent for MNHC collection at the household?
12	What is the best time to collect MNCH data at the household?
13	Do you usually receive support for MNCH surveillance from local health center nurses?
14	Do you usually receive support for MNCH surveillance from Health Zone authorities?

For the nurses, interviews were conducted at HC during working hours. The nurses and their deputies were recruited for the interviews after the obtaining the permission from the KHZ medical officer. Each interview last 30 min. Questions asked to the nurses and local administrative and health authorities are in Table 3.

**Table 3:** Data collection tool for interviews with health and administrative authorities

#	Items
1	The willingness of village leaders, village volunteers, and households to participate in the MNCH surveillance, their capacity and competencies, and the level of accountability of the village committees towards the MNCH community surveillance
2	The appropriate workload to undertake these efforts, the appropriate respondent at the household level, and the best time to collect data at household
3	The appropriate incentives (financial, in-kind, award, and recognition) at the individual or community levels.
4	The coverage of mobile phone services in the villages and the usage of mobile phones in terms of personal and MNCH surveillance-related services
5	Support of provincial and national stakeholders

### Data analysis

Concerning the FGD data, they were simultaneously translated from local languages into French when they were transcribed and then re-read. Analysis of the data was carried out using the framework approach, a method which was developed for systematically analyzing qualitative data [13, 14]. This approach consists of five steps, including familiarization with data, construction of a thematic framework, indexing of the data, and reviewing and summarizing of data [13]. Initially, we analyzed them to highlight the main themes, then we carried out a thematic analysis based on each of these themes: 1) general knowledge of the community-based under-five children healthcare services required and community’s seeking behavior towards these services; 2) community’s practices regarding the seeking behavior for health services for under-five children; 3) community’s attitude towards the mobile phone use and collection of MNCH data, their analysis and use to inform corrective activities; 4) community’s participation in mobile phone data collection, analysis, and use activities; and 5) community’s expectations regarding data collection on the MNCH at the household level. The initial Framework was developed by two investigators following independent reading and joint discussion of the data. Themes that emerged, were shared and discussed with other two investigators prior to summarizing them in the Framework matrices [18].

Software Nvivo 11 was used to analyze data. Data from the FGDs are presented as citations. These citations reflected the understanding and attitudes of households towards the use of mobile phone for the collection, analysis, and use of data for under-five children’s health by VHV. For the data from the structured interviews, we used the usual statistics (proportion, mean, and standard deviation) to describe each group of subjects and present the opinions of the interviewees on the use of the mobile phone for MNCH Surveillance. All data was encoded in Excel 2013 (Microsoft, WA, USA) and analyzed using Stata software version 13.0 (*College Station, TX, USA*).

## Results

### Focus group discussions

#### General knowledge of the community-based under-five children health services

The participants stated that children had health problems, especially fever, respiratory difficulties, malnutrition, and diarrhea due to the lack of good hygiene practice. Vaccinations and distribution of Vitamin A supplementation and deworming tablets were important services provided to children. They cited the following:

*“Lately, the children suffered from diarrhea, high fever, and loss of blood and water. Very often, I saw and learned about an increasing number of children receiving blood and water at the hospital. People do not understand. There are many children and mothers who die in our community.”*

“*We talk about the epidemic of malaria, which requires infusion and transfusion. In the case of malaria, nurses consult our children and give quinine. However, after two weeks, we observed again high fever in children.”*

*“We have mosquito nets in our homes, but our children die. We do not have the peace and desire to have children. Last time, the epidemic of fever for children lasted two months and we knew nothing. Health workers must do something to detect this disease. The future of our children is in jeopardy.”*

In expounding, FGD participants reported that fever also had subtle cultural and beliefs, such as the involvement of witchcraft, especially when the fever did not disappear even after the child had received treatments at the HC.

*“We use long acting insecticide bed nets and take treatments at health centers, but our children were still suffering from the fever. You see, there are people who practice witchcraft in this area.”*

Poor hygiene has been identified as the main cause of diarrhea and disease. The participants expressed this in the following terms:

*“Mothers wash vegetables with dirty water. All this causes disease. We are not used to boiling water before consuming it. We drink it as it is. Maybe this causes diarrhea to our children.”*

*“Women breastfeed without washing the breasts. When they come from the farm, children suck breasts that contain sweat. When women use the toilet, they put their hands directly in the food without washing them.”*

*“Poor hygiene and lack of toilets in the villages make children sick. They have worms. All children wash in the same water basin in turn, you will see even 5 children wash their hands in one place and distribute microbes. Regarding the use of latrines or cabinets, we see a village of 50 households with only 10 toilets. Other people go to the forest for defecation, saying that pigs will eat it. When children go to play, they walk on feces that contain hookworms, and even if they have received mebendazole during the campaign, hookworms will remain in their bodies. Children cannot be healthy. This problem is due to the negligence and lack of toilets.”*

Malnutrition has also been identified as a health problem among young children and this situation was more prominent when the mothers were expecting other children. This view was cited as follows:

*“Children are not in good health. They refuse to eat; the tummy becomes big and they do not play with others. I see that this situation happens when mothers are pregnant and they do not have energy to give children good food.”*

#### Community practices regarding the seeking behavior for health services for under-five children

Households were aware of health issues and usually sought medical attention for their children at HCs when they fell sick or required vaccinations.

*“When a child falls ill, it is brought to the HC for treatment and blood tests. Nurses give medications and parents go back home.”*

Participants claimed that the parents must take care of their children and that the importance of giving medical attention to the child has been emphasized to them by the healthcare workers. This was stated in the following terms:

*“We need to take better care of our children from birth until they are 5 years old. Experts tell us that prevention is better than cure. We also follow the advice on the radio about bringing our children to health centers for vaccinations, vitamins, and mebendazole.”*

However, there were local issues that have been identified as barriers to the access of care. The participants explained that many households were struggling with unique socio-economic barriers to care. This resulted in delaying in seeking medical attention.

*“Children are dying due to neglect. They can have high fever for 2-3 days, but if the mother has no money to bring him to the hospital, she will stay at home and only give aspirin and vitamin. And when the child's condition worsens, it is then taken to the hospital for transfusion and infusion, and this situation can sometimes result in death.”*

Beliefs and cultural norms contribute to the practices of seeking health services. The community was facing a dilemma between choosing parallel healthcare providers and community health workers (CHW) for their under-five children. In the search of complying with their cultural belief, it was asserted that many parents choose a traditional healthcare provider, even though sufficient numbers of CHWs were available. This often led to parents seeking care from healthcare providers who may lack expertise in under-five children clinical management. This situation was mentioned by the participants. One man said the following:

*“Many mothers trust traditional healthcare providers, who are not nurses. If you have no money, you can give them chicken, cassava, beans, or corns that you have in the house and they provide the treatment. But, these people are charlatans in my view. In a few cases, they can treat well the child, but in most of time, parents are obliged to take the sick child to the hospital after wasting their time and belongings on these charlatans.”*

In addition, the quality of services provided by CHW came under scrutiny. Participants said that CHWs were trained, but they were not nurses. This was expressed in the following terms:

*“CHWs began to treat children and when the disease gets complicated, and it is often too late.”*

*“CHWs do not always treat the children well. They must study to become nurses. I know a case of my neighbor’s child. This child was treated at home by a CHW until the disease complicated so that he could be referred to the HC. But it was too late because the child died.”*

Another issue related to the seeking behavior for under-five children health services was religion. Some religion and the new churches have a negative attitude towards health of children, as the participants cited:

*“I am in the community health group accompanying nurses for vaccination since 2012. The black church believers do not agree to have their children vaccinated. Some of them call it “delayed poison” because when children receive the vaccine, they get sick with fever. However, we all know that if the child gets a fever after vaccination, we must give paracetamol. But the people do not want this. They say that they grew up without taking vaccines.”*

Households knew the health conditions of under-five children in the communities. They cited mainly diarrhea, dehydration, high fever, malnutrition and related campaigns for the distribution of Vitamin A and deworming:

*“There is the presence of several illnesses in children lacking food before 6 months. Children suffered from diarrhea, high fever, loss of blood and water.”*

#### Community’s attitude towards the use of the mobile phone use and collection of data

The advantages of mobile phones were numerous and very visible in the communities. Mobile phone was perceived as a device that helps render services quickly for people who need help.

*“The phone has the advantage of making emergency calls in case of sickness and ask for medications and materials instead of going to the hospital, which might be too late if you do not have money. The device is faster to send messages and data.”*

The FGDs indicated the impact of mobile phones on daily lives of those who had them. They received money and goods from their families in town and communicated easily with nurses about the problems that their children and wives may have. Two women said:

*“This phone made other people rich. They can receive money and get assistance when needed from other people. It is pity for those who do not have them.”*

*“I like the mobile phone application so much. One day, my neighbor just called his daughter from Kinshasa. The following day, he received money from a number in the mobile phone. He took this number to a shop in our village and they gave him money. This was good and easy.”*

However, mobile phones lead to additional expenses. Such economic consequences can be very far reaching and eventually disrupt the peace of the family. One woman said:

*“You must buy call credits every time and pay for changing them. Even if you do not call, the charge of the phone decreases. Instead of buying food, people buy phone credits and there are fights in the house.”*

#### Community’s participation in mobile phone data collection, analysis, and use activities

The collection of data using mobile phone is very common. One woman said:

*“We saw some people from Kinshasa using the phone to ask questions about usage of toilet.”*

The role of household in collecting data is limited to only providing answers to questions asked by those conducting these activities. One woman said:

*“We always listen to the questions and give answers. The person who asks the questions just presses the button in the mobile phone.”*

People were sometimes impressed by this device as it registered the answers, and no one needs to writing anything. One man said:

*“We see the people with these phones. Instead of calling and talking, they just ask questions and press the button. It is cool. There is no paper and writing involved.”*

In relation to the lack of financial assistance to households, participants stressed that CHWs and VHV data collectors were well treated by the government, with a disproportionate amount of benefits ranging from free healthcare at HCs to direct financial incentives from donors.

*“These people do not give us anything. We saw them having a mobile phone and buying nice foods whilst we, who answered their questions, have nothing. This is not fair to us.”*

However, although this was rarely mentioned in the FGDs, data collection using mobile phone has been mentioned as means by which data collectors make money and get health advantages without making the effort of writing on paper. Households who provide the information to them have nothing, even not free healthcare beside vaccinations. It was expressed that more households would become involved in MNCH activities if they were more aware of the linkages to better health outcome of their children, even if there are no financial reimbursement.

*“We will always participate in answering questions even if these people do not give us anything.”*

*“If, these activities help us in protecting our children from getting sick and our wives dying of pregnancy, we will participate in answering questions and discuss on how to improve our health.”*

Additionally, it was discussed that households would be more attractive to MNCH activities using mobile phone if they stayed abreast of the complexities of health services for children.

*“For example, women breastfeed without washing the breasts. When they come from the field, children suck breasts that contain sweat and dirt. This situation can be studied and discussed among us so that all women stop doing it.”*

#### Community’s expectations regarding data collection on MNCH at the household level

The low attendance rates during data collection activities on MNCH conducted so far by the VHV in the KHZ is of serious concern. When discussing about the expectations regarding data collection on MNCH at the household level during the FGDs, three main themes emerged: (1) expectation of free health services for their children; (2) material benefits for households; and (3) no obvious links observed between the activities of data collection on MNCH surveillance and the health benefits for under-five children.

Lack of incentives for households was been mentioned increasingly during the discussions. One woman and man said:

“*We do not receive anything from these people who visit our houses, except medications against worms, vitamins, and vaccines for our children. We do not get anything.*”

*“They do not explain to us why they are asking questions. They just tell us to answer to the questions. We know that receive money but nothing is given to us.”*

*“They are just asking questions, but do not bring anything for the children like foods or toys.”*

Data collectors do not explain the purpose of collecting data and how it may benefit them. One man said:

*“We would like to know what they are doing with the data they are collecting. One day, I asked one data collector this question: why are you always asking us questions about the health of our children. He told me that the doctor has asked for it. You see, maybe the doctor wants this information so that he sends it to Kinshasa and receive more money from Kinshasa for himself.”*

People should know why data for their under-five children has been collected. Lack of explanation about the use of these data for the household is a gap in MNCH surveillance.

At least one group mentioned the necessity of data collection for better planning for health services for children. One man said:

*“We assume that the information we give to the VHV is sent to the doctor and authority. Then, they provide vaccines, vitamins, and medications against worms to our children. That it is why I always say that it is good to give them the information about the health of our children.”*

### Structured interviews

#### Village health volunteers

All the VHV interviewed (190) were mostly adult men [mean age 46 years (SD = 6 years); minimum = 35; maximum = 56] and women [mean age 44 years (SD = 6); minimum = 30; maximum = 53] and6 had completed middle (48%) and high school (52%) education. All married men were involved in some income-generating activities, such as agriculture (77%), fishing (19%), and hunting (4%). Women represented 5% (10) of the VHV participants. Most of these women (8) were involved in agriculture and small business. All participants had been living in the present villages for some time ranging between 20 and 45 years.

In Table 4, 91.1% (173/190) participants were mentioned willing to participate in MNCH surveillance. Prior to the study, only 72.1% estimated having the capacity and competence for using mobile phone for MNCH surveillance, 17.9% expressed the need to be trained more, and 10% wanted additional assistance to become familiar with the technology. These workers estimated that around five households could be surveyed in a day; the actual number varied from 3 to 15 households. More than 5, 6–10, and 11–15 households were surveyed by 64.2%, 29%, and 6.3% of the VHV. Regarding the willingness to undertake MNCH activities using mobile phones, 72.1% VHV felt that they could do the job, 17.4% felt compelled to do it, and 10.5% felt that it was someone else’s job (Table 4). The possibility of receiving incentives for the MNCH surveillance using mobile technology was most welcomed, however, the expected incentives were mainly financial (48.4%), followed by the expectation of gift in the form of the mobile phone (17.9%) used for MNCH activities, an award (14.2%), free healthcare for the family (12.1%), public recognition (4.2%), and gifts in kind for the community (3.2%). Most VHV estimated that the appropriate respondents at the household were mothers (73.7%); followed by fathers (8.4%), grand-parents (7.9%), aunts (5.8%), and uncles (4.2%). These VHV estimated that the most appropriate time for data collection was noon during the weekdays (63.2%), followed by the evening (34.2%), afternoon (2.1%), and morning (0.5%).

**Table 4:** Willingness, commitment, capacity and competencies, and motivation to perform the MNCH surveillance

Variables	Nbr (*n* = 190)	%
Willingness to participate in the MNCH surveillance	173	91,1
Having the capacity and competencies to use mobile phone for surveillance		
Able to use mobile phones	137	72,1
Need to be trained more	34	17,9
Need additional assistance to become familiar with the technology	19	10,0
Estimates for daily workload in collecting data (in households)		
≤ 5	122	64,2
6–10	56	29,5
11–15	12	6,3
Willingness to undertake MNCH activities using mobile phones		
Willingness to do the work	137	72,1
Recognizing that this is their job	33	17,4
Recognizing that this is an optional work	20	10,5
Expected incentives		
Financial	92	48,4
Gift of the mobile phone	34	17,9
An award	27	14,2
Free healthcare for the family	23	12,1
Public recognition	8	4,2
In-kind gift for the community	6	3,2
Appropriate responders at household		
Mothers	140	73,7
Fathers	16	8,4
Grand-parents	15	7,9
Aunts	11	5,8
Uncles	8	4,2
Timing for collecting data		
Noon time during the weekdays	120	63,2
Evening	65	34,2
Afternoon	4	2,1
Morning	1	0,5

**Fig. 1:**
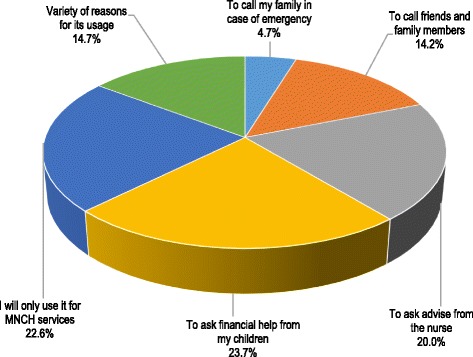
The usage of mobile phone in terms of personal and MNCH surveillance-related services

**Fig. 2:**
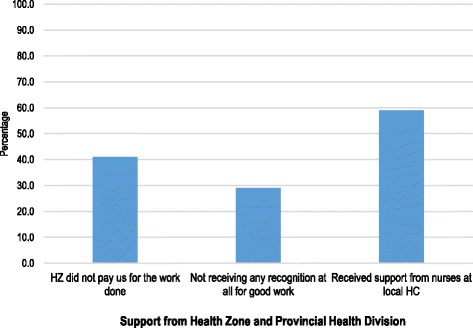
Support from Provincial Heath Division and Health Zone Management

Figure 1 shows the usage of the mobile phones; only 22.6% VHV recognized that the device should be used only for MNCH surveillance. Other usages included: asking for financial help from their children (23.7%), asking for advice from the local nurse (20.0%), to call friends and family members (14.2%), other reasons (14.7%), and to call family members in case of emergency (4.7%). In Fig. 2, just below half of VHV (41.1%) estimated that the HZ did not pay them for the work done, 29.0% felt that they had not received any recognition for the good work so far, and 58.9% received support from the local HC nurses.

#### Nurses at the local health centers

We interviewed five nurses, three men and two women [mean age 42 years (DS = 6 years)]. All of them had been working in these areas for 4 to 20 years. They were married, with an average of two children aged below 5 years. All these nurses supervised local VHV for quarterly report and distribution of iCCM related products and vaccinations. All nurses estimated that the VHV were willing to participate in the MNCH surveillance using mobile phones and that they could perform competently if trained well. Four out of five nurses suggested that the VHV were accountable for the MNCH surveillance, but regular reminders and follow-ups were needed. The appropriate workload to undertake these efforts were estimated to be between 5 to 10 households per day, considering the distance between the households within the villages; and that the appropriate respondents at the household level would be the mothers; and the best time to collect data would be in the afternoon (80%) or during the day on Sundays (20%). Nurses estimated the appropriate incentives to be more financial (60%), followed by in-kind (27%), and for the individual and in-kind at the community level (13%). The coverage of mobile phone services in the villages had been estimated to be good in the eastern area and average in the western part of the KHZ. Nurses declared that the VHV will also use the donated mobile phone for personal use, because it usually increases the social status in the community. They thought that the VHV enjoyed their full support and occasional support from the HZ office, and that provincial and national health administration structures were not adequate to be involved at this level of activities.

#### Local administrative and health authorities

The five staff members interviewed from the KHZ Administrative and Health Authority were all adult men (mean age 53 years). They had been working in the KHZ for 5 to 18 years. All of them were married with an average of two children aged below 5 years. The Local Authority supervises the overall administration of health and civic activities at village level. The interviews indicated the following: All administrative staff estimated that the VHV were willing to participate in the MNCH surveillance using mobile phones and that they could perform competently if trained well. Four out of five administrative staff suggested that the VHV were accountable for the MNCH surveillance, because they were already doing the work and the mobile phone would help facilitate it.

The appropriate workload to undertake these efforts was estimated between 10 to 15 households per day considering the distance between the households within the villages; the appropriate respondents at the household level would be the mothers (80%) or grand-mothers (20%); and the best time to collect data at the household would be late in the afternoon (40%) or during the day on Sundays (60%). Regarding incentives, three nurses estimated the appropriate incentives to be more financial; in-kind at individual and in-kind at community levels were estimated to be appropriate incentives by one nurse each. The coverage of mobile phone services in the villages was been estimated to be good for one cellphone network, and not so good for the others. They believed that the mobile phones would be used by the VHV for all purposes, including personal needs. All the administrators thought that the VHV would need more support from the HZ, especially while developing corrective actions, and that the health activities at this level were beyond the scope of the provincial health administration.

## Discussion

The objective of this study was to determine the perceptions of households, attitudes of community health volunteers, and opinions of nurses in HC and administrative authorities towards the use of mobile phones for MNCH surveillance in the rural KHZ in the DRC.

The results shown that there were strong and positives perceptions for the use of mobile phone for the MNCH surveillance to strengthen health services for mothers, newborn, and children in the remote area of DRC, where health indicators were the most of concern. This study described the local stakeholders’ perceptions to mobile phones in the health context of surveillance and their use by local VHV, who play a major role in filling gaps for community health. The perceptions of households and local health service providers were critical for the success implementation of this surveillance. Most critical issue was the acceptance by VHV to use the mobile phone for MNCH surveillance. Majority of participants in the discussions have knowledge of benefits of mobile phone in terms of communication and quick response to health-related assistance.

It is of interest that the household’s FGD’s revealed the knowledge of the MNCH surveillance activities and that the use of mobile phone technology, and the faith in the VHV to collect data for their children’s health issues. The participants in these discussions believed that effective supervision and training for the VHV would include components to acquire knowledge of mobile phone application, and data analysis, and use. Such training programs would serve to equip the VHV with technical competency for the use of mobile phone, and more importantly with the use of the data that will be collected. It also was expressed that the VHV will need assistance from nurses and local health authorities in the data analysis, use, and formulation of action to improve the health situation of their children in the villages.

Direct benefits from the implementation of the activities of MNCH surveillance using mobile phone for children health were found by participants in the discussions to be the most expected benefits of these efforts. These findings were in line with a similar study conducted in Zambia [19]. In addition, as shown by other investigators [19–21], households have a general knowledge of the community-based under-five children health issues, and the services and that the seeking behavior was mainly influenced by financial and geographic challenges. Moreover, these challenges oblige many households to use alternative care mainly due to the cultural and beliefs as shown in other studies [22, 23]. Like other investigators [24], this study has shown that households recognized fever, diarrhea, and chest infections (difficulty in respiration) as the most common causes of children health issues. This study has found that the community’s expectations were very high regarding data collection on MNCH at the household level. Most of the group discussions pointed out to the need for better health for their children, which will reduce the expenditure on healthcare services.

The VHV acceptance to use of mobile phone for the collection of data detailed in this study were like those in other studies conducted at community level [25–30]. There was high willingness among these VHV to use mobile phone for collection and use of data at household’s level. This was like a study conducted by Kazi et al. in Kenya, where respondents from rural were willing to use mobile phone through text messages for MNCH interventions [31].

Motivation to accept undertake these activities were perceived as important factor to all VHV and households. These incentives were estimated to be provided at the individuals and be mainly financial. This study found that VHV were willing to participate in the MNCH surveillance because the use of mobile phone improve MNCH surveillance compared to paper-based task as shown by other investigators [32, 33]. Despite the need for financial incentives for individuals rather than for the community, the participants agreed that most of the VHV were accountable and could competently collect data using mobile phones if they were trained well. Financial incentives for individuals have been found a limiting factor in the implementation of intervention at community level in many settings [34–36]. However, this barrier can be overcome if the intervention produces a desire for self-development and the improvement of community health, and utilization of free time are met. These elements may be a sustainable solution for the implementing of such intervention by volunteers [35, 36].

Furthermore, the use of these communication devices by the VHV for personal needs was very certain because of the high social status that these devices provide to the holder.

However, there are certain limitations to this study. The summaries of the FGDs were developed using independent transcription processes and quantitative measurements to code or rate the outcomes; the investigators developed narrative summaries based on the review of audio tape recordings and written notes of the discussions. This may have introduced a subjective bias, thereby posing a reliability threat to the discussion findings. However, this limitation is believed to be remote, since there has been double entry of data and coding. Moreover, the information from the FGDs and interviews of the VHV, local nurses, and administrative authority converges with the other findings from previous investigators.

Regarding the quantitative approach, it was not easy to determine to what extent the interview on the use of mobile phones for MNCH surveillance could have influenced the responses from the interviewees. In a sense, these responses may reflect the intention of respondents to get refilling airtime for mobile phones, in addition to getting a new mobile phone if it was provided or another mobile phone if the respondents already had one.

## Conclusion

In conclusion, health issues and services for under-five children are well known by households, who estimated that VHV could use mobile phone to collect MNCH data and that local nurses could play a supervisory role. The children’s mothers were viewed as best respondents at household level. Furthermore, all local stakeholders estimated VHV to be able to use mobile phone for collection and analysis of MNCH data. The VHV themselves were willing to use mobile phone for MNCH surveillance activities from which they were expecting to gain benefits.

## Additional file

Additional file 1: Selected villages for VHV interviews. (DOCX 13 kb)

## Abbreviations

ACT: Artemissin combination treatment
CHW: Community health workers
DHI2: District Health Information System 2
DRC: Democratic Republic of the Congo
FGD: Focus group discussion
HC: Health centers
HZ: Health Zone
iCCM: Integrated community case management
KHZ: Kenge Health Zone
MNCH: Maternal Newborn and Child Health
SD: Standard deviation
U5MR: Under-five mortality rate
VHV: Village health volunteers
WHO ERC: World Health Organization Research Ethics Review Committee
