# Supplementary material for: Community-based maternal, newborn, and child health surveillance: perceptions and attitudes of local stakeholders towards using mobile phone by village health volunteers in the Kenge Health Zone, Democratic Republic of Congo
Source: BMC Public Health. 2018 Mar 5;18:316. doi: 10.1186/s12889-018-5186-2 (PMC5838964; doi:10.1186/s12889-018-5186-2)
Supplement: Supplementary file 1 — Selected villages for VHV interviews. (DOCX 13 kb) [file 12889_2018_5186_MOESM1_ESM.docx]

| **Selected villages for VHV interviews** | | | | |
| --- | --- | --- | --- | --- |
| Basensi | Ifumu | Kimbubu Loko | Luzau | Mukondo-Tuana |
| Bimilu Kalenge | Ilesi-Malungu | Kimbumba II | Mabaka | Mukukulu –Tseke |
| Bis-a-Bisi | Impaka | Kimwabi | Mabaka-Wembo | Mulombi |
| Bisima | Indima | Kimwanza | Mabemba | Mupumbu |
| Bitadi-Lonzo | Ingoy | Kina-Yamvu | Makaya | Musenga-Mbau |
| Bitumbu | Iniangi | Kindombi | Makongo | Mutete II |
| Bivuanda | Inzioko | Kindundu | Makwati | Muzengo |
| Buka-Kitubu | Inzita | Kingoma | Malombala | N’Koku |
| Bukanga- Mofete | Itswadi | Kinzofo | Mandinda | Ngla Kutu |
| Bukanga-Nzadi | Ipandi | Kipalanga Basin | Manunga Plantation | Ngunga |
| Bumbi | Kalengi | Kisangi | Masabu | Nto-Pemba |
| Epeya | Kalunga Bidi | Kisiamasia | Masebo | Nzasi |
| Fakamba | Kapanga | Kisimuna-Lono | Mawowa | Pasanzi |
| Ferme Mwanza | Kapay-Lwono | Kitaka Tsay | Mayeno | Pont – Kwango |
| Foreami | Kibiniti-Nga | Kitotila Tseke | Mbeko | Siangolo |
| Ibayila | Kikuti | Lukungu | Mbinda-Dinga | Swa-Ikemba |
| Ihadi-Mpungu | Kikwanga | Lumwambu | Mbinda-Pumbu | Sweka Kitambo |
| Ikabanga | Kimbanzi | Luntaleti | Mtsakala | Tavunda |
| Ikawu | Kimbinda | Luwaku | Muela Masingi | Tsakala-Bukanga |
